# Supplementary figures and images for: Banned by the law, practiced by the society: The study of factors associated with dowry payments among adolescent girls in Uttar Pradesh and Bihar, India
Source: PLoS One. 2021 Oct 15;16(10):e0258656. doi: 10.1371/journal.pone.0258656 (PMC8519446; doi:10.1371/journal.pone.0258656)

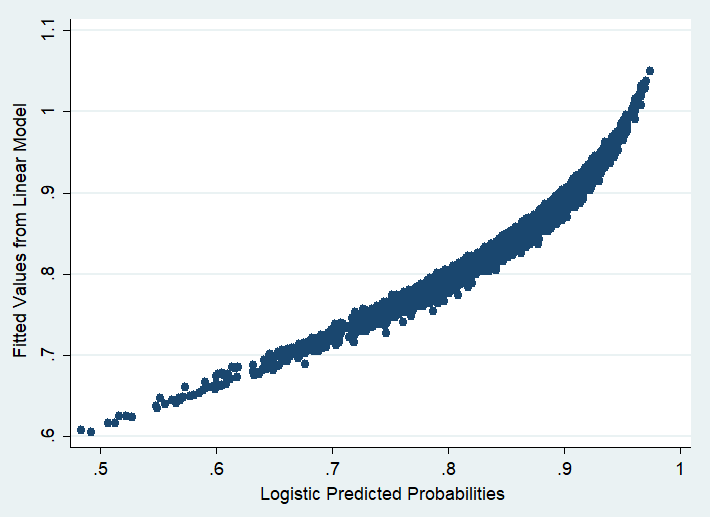


**Figure-S1**. Plot of logistic predicted probabilities vs linear model

Supplement: S1 Fig — (DOCX) [file pone.0258656.s001.docx]

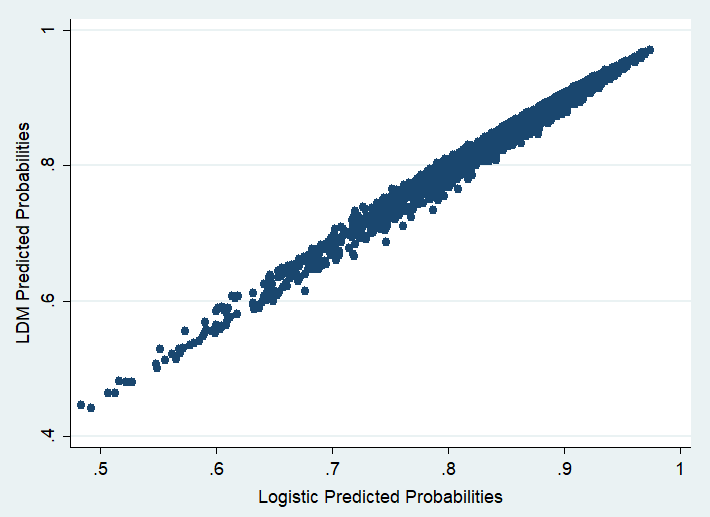


**Figure-S2.** Plot of logistic predicted probabilities vs LPM.

Supplement: S2 Fig — (DOCX) [file pone.0258656.s002.docx]
