# Supplementary material for: Banned by the law, practiced by the society: The study of factors associated with dowry payments among adolescent girls in Uttar Pradesh and Bihar, India
Source: PLoS One. 2021 Oct 15;16(10):e0258656. doi: 10.1371/journal.pone.0258656 (PMC8519446; doi:10.1371/journal.pone.0258656)
Supplement: S1 Table — (DOCX) [file pone.0258656.s003.docx]

| **Table-S1.** Logistic regression estimates for adolescents who paid dowry by background characteristics (15-19 years) | | |
| --- | --- | --- |
| **Variable** | **Urban [OR (CI)]** | **Rural [OR (CI)]** |
| **Husband known before marriage** |  |  |
| Not Known | Ref. | Ref. |
| Known | 0.40*(0.31,0.51) | 0.64*(0.51,0.81) |
| **Age at marriage** |  |  |
| Less than legal age | Ref. | Ref. |
| More than legal age | 1.48*(1.04,2.1) | 1.63*(1.14,2.32) |
| **Spousal age gap** |  |  |
| Wife older/almost same age | Ref. | Ref. |
| Husband older | 1.57(0.99,2.49) | 1.15(0.78,1.68) |
| **Spousal education** |  |  |
| Both not educated | Ref. | Ref. |
| Only husband educated | 1.29(0.81,2.06) | 1.47*(1.03,2.09) |
| Only wife educated | 1.61*(1.04,2.5) | 1.34(0.92,1.95) |
| Both educated | 1.51*(1.05,2.18) | 2.15*(1.56,2.97) |
| **Working status** |  |  |
| No | Ref. | Ref. |
| Yes | 0.98(0.65,1.47) | 0.75(0.56,1.01) |
| **Vocational training received** |  |  |
| Not received | Ref. | Ref. |
| Received | 0.94(0.68,1.31) | 1.51*(1.05,2.16) |
| **Mother education (in years)** |  |  |
| No education | Ref. | Ref. |
| 1-7 | 1.05(0.66,1.69) | 0.93(0.62,1.41) |
| 8-9 | 1.21(0.65,2.25) | 1.58(0.81,3.07) |
| 10 and above | 0.44*(0.27,0.73) | 1.17(0.59,2.3) |
| **In-laws land ownership** |  |  |
| No | Ref. | Ref. |
| Yes | 1.17(0.78,1.75) | 0.98(0.77,1.26) |
| **Caste** |  |  |
| SC/ST | Ref. | Ref. |
| Non-SC/ST | 1.15(0.86,1.55) | 1.01(0.79,1.28) |
| **Religion** |  |  |
| Hindu | Ref. | Ref. |
| Non-Hindu | 0.92(0.66,1.27) | 0.95(0.68,1.32) |
| **Wealth index** |  |  |
| Poorest | Ref. | Ref. |
| Poorer | 1.48(0.87,2.51) | 1(0.74,1.34) |
| Middle | 1.59(1.00,2.52) | 1.27(0.92,1.77) |
| Richer | 1.77*(1.12,2.78) | 1.32(0.91,1.92) |
| Richest | 2.02*(1.22,3.34) | 1.42(0.87,2.31) |
| **State** |  |  |
| Uttar Pradesh | Ref. | Ref. |
| Bihar | 1.35*(1.02,1.78) | 1.48*(1.17,1.87) |

*if p<0.05, Ref: Reference; OR: Odds Ratio; CI: Confidence Interval; SC/ST: Scheduled Caste/Scheduled Tribe; Not legal age: less than 18 years; Legal age: More than 18 years
