# Supplementary material for: Banned by the law, practiced by the society: The study of factors associated with dowry payments among adolescent girls in Uttar Pradesh and Bihar, India
Source: PLoS One. 2021 Oct 15;16(10):e0258656. doi: 10.1371/journal.pone.0258656 (PMC8519446; doi:10.1371/journal.pone.0258656)
Supplement: S2 Table — (DOCX) [file pone.0258656.s004.docx]

| **Table-S2.** Percentage distribution of adolescents who paid dowry by region, 15-19 years | | | | |
| --- | --- | --- | --- | --- |
| **Variable** | **Paid dowry** | | | |
|  | Uttar Pradesh | p-value | Bihar | p-value |
| **Husband known before marriage** |  | * |  | * |
| Not known | 85.1 |  | 89.7 |  |
| Known | 83.4 |  | 81.4 |  |
| **Age at marriage** |  | * |  | * |
| Less than legal age | 82.9 |  | 87.3 |  |
| More than legal age | 88.4 |  | 92.1 |  |
| **Spousal age gap** |  |  |  |  |
| Wife older/almost same age | 83.9 |  | 86.7 |  |
| Husband older | 84.4 |  | 87.8 |  |
| **Spousal education** |  | * |  | * |
| Both not educated | 73.7 |  | 80.8 |  |
| Only husband educated | 79.8 |  | 86.4 |  |
| Only wife educated | 82.8 |  | 84.6 |  |
| Both educated | 86.6 |  | 90.8 |  |
| **Working status** |  |  |  | * |
| No | 84.4 |  | 88.5 |  |
| Yes | 84.3 |  | 81.4 |  |
| **Vocational training received** |  | * |  | * |
| Not received | 83.2 |  | 87.1 |  |
| Received | 91.1 |  | 91.1 |  |
| **Mother education (in years)** |  | * |  | * |
| No education | 83.7 |  | 87.4 |  |
| 1-7 | 88.5 |  | 84.9 |  |
| 8-9 | 86.5 |  | 96.8 |  |
| 10 and above | 87.9 |  | 90.9 |  |
| **In-laws land ownership** |  | * |  | * |
| No | 82.9 |  | 86.3 |  |
| Yes | 86.0 |  | 90.6 |  |
| **Caste** |  |  |  | * |
| SC/ST | 84.7 |  | 86.6 |  |
| Non-SC/ST | 84.2 |  | 88.2 |  |
| **Religion** |  |  |  | * |
| Hindu | 84.4 |  | 88.1 |  |
| Non-Hindu | 84.2 |  | 85.4 |  |
| **Wealth index** |  | * |  | * |
| Poorest | 75.7 |  | 86.6 |  |
| Poorer | 81.4 |  | 85.9 |  |
| Middle | 85.4 |  | 89.0 |  |
| Richer | 84.8 |  | 90.2 |  |
| Richest | 89.3 |  | 85.7 |  |
| **Place of residence** |  | * |  | * |
| Urban | 77.0 |  | 83.9 |  |
| Rural | 85.9 |  | 88.1 |  |
| **Total** | **84.4** |  | **87.8** |  |

*if p<0.05; SC/ST: Scheduled Caste/Scheduled Tribe; Not legal age: less than 18 years; Legal age: More than 18 years
