# Supplementary material for: Banned by the law, practiced by the society: The study of factors associated with dowry payments among adolescent girls in Uttar Pradesh and Bihar, India
Source: PLoS One. 2021 Oct 15;16(10):e0258656. doi: 10.1371/journal.pone.0258656 (PMC8519446; doi:10.1371/journal.pone.0258656)
Supplement: S3 Table — (DOCX) [file pone.0258656.s005.docx]

| **Table-S3.** Stepwise logistic regression estimates for adolescents who paid dowry by background characteristics (15-19 years) | | | | | | |
| --- | --- | --- | --- | --- | --- | --- |
| **Variable** | **Model-1 [OR (CI)]** | **Model-1 [OR (CI)]** | **Model-1 [OR (CI)]** | **Model-1 [OR (CI)]** | **Model-1 [OR (CI)]** | **Model-1 [OR (CI)]** |
| **Husband known before marriage** |  |  |  |  |  |  |
| Not Known | Ref. |  |  | Ref. |  | Ref. |
| Known | 0.49***(0.41 -0.57) |  |  | 0.49***(0.41 -0.57) |  | 0.52***(0.44 -0.61) |
| **Age at marriage** |  |  |  |  |  |  |
| Less than legal age | Ref. |  |  | Ref. |  | Ref. |
| More than legal age | 1.43***(1.13 -1.82) |  |  | 1.44***(1.13 -1.83) |  | 1.52***(1.19 -1.95) |
| **Spousal age gap** |  |  |  |  |  |  |
| Wife older/almost same age | Ref. |  |  | Ref. |  | Ref. |
| Husband older | 1.28*(0.96 -1.7) |  |  | 1.30*(0.97 -1.73) |  | 1.27(0.95 -1.69) |
| **Spousal education** |  |  |  |  |  |  |
| Both not educated | Ref. |  |  | Ref. |  | Ref. |
| Only husband educated | 1.49***(1.13 -1.96) |  |  | 1.43**(1.09 -1.88) |  | 1.41**(1.07 -1.86) |
| Only wife educated | 1.54***(1.17 -2.04) |  |  | 1.45***(1.1 -1.93) |  | 1.5***(1.13 -1.99) |
| Both educated | 2.13***(1.72 -2.65) |  |  | 1.93***(1.54 -2.43) |  | 1.91***(1.51 -2.41) |
| **Working status** |  |  |  |  |  |  |
| No |  | Ref. |  | Ref. | Ref. |  |
| Yes |  | 0.73***(0.58 -0.91) |  | 0.78**(0.62 -0.99) | 0.77**(0.61 -0.98) |  |
| **Vocational training received** |  |  |  |  |  |  |
| Not received |  | Ref. |  | Ref. | Ref. |  |
| Received |  | 1.24*(0.99 -1.57) |  | 1.21(0.96 -1.53) | 1.20(0.95 -1.52) |  |
| **Mother education (in years)** |  |  |  |  |  |  |
| No education |  | Ref. |  | Ref. | Ref. |  |
| 1-7 |  | 1.14(0.85 -1.54) |  | 1.03(0.76 -1.4) | 1.08(0.79 -1.45) |  |
| 8-9 |  | 1.53*(0.99 -2.38) |  | 1.45(0.93 -2.27) | 1.44(0.92 -2.24) |  |
| 10 and above |  | 0.76(0.53 -1.1) |  | 0.73*(0.50 -1.06) | 0.66**(0.45 -0.96) |  |
| **In-laws land ownership** |  |  |  |  |  |  |
| No |  | Ref. |  | Ref. | Ref. |  |
| Yes |  | 1.35***(1.13 -1.61) |  | 1.21**(1.01 -1.45) | 1.11(0.90 -1.35) |  |
| **Caste** |  |  |  |  |  |  |
| SC/ST |  |  | Ref. |  | Ref. | Ref. |
| Non-SC/ST |  |  | 1.15(0.96 -1.38) |  | 1.10(0.92 -1.33) | 1.09(0.91 -1.31) |
| **Religion** |  |  |  |  |  |  |
| Hindu |  |  | Ref. |  | Ref. | Ref. |
| Non-Hindu |  |  | 0.81*(0.66 -1.01) |  | 0.83(0.67 -1.04) | 0.95(0.76 -1.18) |
| **Wealth index** |  |  |  |  |  |  |
| Poorest |  |  | Ref. |  | Ref. | Ref. |
| Poorer |  |  | 1.20(0.94 -1.54) |  | 1.14(0.88 -1.46) | 1.13(0.88 -1.45) |
| Middle |  |  | 1.53***(1.20 -1.96) |  | 1.43***(1.11 -1.85) | 1.40**(1.08 -1.80) |
| Richer |  |  | 1.67***(1.30 -2.15) |  | 1.54***(1.18 -2.00) | 1.50***(1.15 -1.94) |
| Richest |  |  | 1.88***(1.41 -2.52) |  | 1.73***(1.27 -2.37) | 1.56***(1.15 -2.13) |
| **Place of residence** |  |  |  |  |  |  |
| Urban |  |  | Ref. |  | Ref. | Ref. |
| Rural |  |  | 1.67***(1.41 -1.98) |  | 1.62***(1.35 -1.94) | 1.54***(1.29 -1.83) |
| **State** |  |  |  |  |  |  |
| Uttar Pradesh |  |  | Ref. |  | Ref. | Ref. |
| Bihar |  |  | 1.40***(1.19 -1.65) |  | 1.42***(1.2 -1.68) | 1.39***(1.17 -1.65) |
| ***if p<0.001; **if p<0.05; *if p<0.10; Ref: Reference; OR: Odds Ratio; CI: Confidence Interval; SC/ST: Scheduled Caste/Scheduled Tribe; Not legal age: less than 18 years; Legal age: More than 18 years | | | | | | |
