# Supplementary material for: Banned by the law, practiced by the society: The study of factors associated with dowry payments among adolescent girls in Uttar Pradesh and Bihar, India
Source: PLoS One. 2021 Oct 15;16(10):e0258656. doi: 10.1371/journal.pone.0258656 (PMC8519446; doi:10.1371/journal.pone.0258656)
Supplement: S4 Table — (DOCX) [file pone.0258656.s006.docx]

| **Table-S4** Summary statistics for LPM. | | | | | |
| --- | --- | --- | --- | --- | --- |
| Variable | Obs | Mean | Std. Dev. | Min | Max |
| yhat | 5,206 | 0.84 | 0.07 | 0.61 | 1.05 |
| yhat_ldm | 5,206 | 0.84 | 0.08 | 0.44 | 0.97 |
| yhat_logit | 5,206 | 0.84 | 0.07 | 0.48 | 0.97 |

Obs: Observation; Std. Dev.: Standard Deviation; Min: Minimum; Max: Maximum
