# Supplementary material for: Banned by the law, practiced by the society: The study of factors associated with dowry payments among adolescent girls in Uttar Pradesh and Bihar, India
Source: PLoS One. 2021 Oct 15;16(10):e0258656. doi: 10.1371/journal.pone.0258656 (PMC8519446; doi:10.1371/journal.pone.0258656)
Supplement: S5 Table — (DOCX) [file pone.0258656.s007.docx]

| **Table-S5** Correlation index for robustness check of LPM | | | |
| --- | --- | --- | --- |
|  | yhat | yhat_ldm | yhat_logit |
| yhat | 1 |  |  |
| yhat_ldm | 0.972 | 1 |  |
| yhat_logit | 0.975 | 0.994 | 1 |
