# Supplementary material for: Banned by the law, practiced by the society: The study of factors associated with dowry payments among adolescent girls in Uttar Pradesh and Bihar, India
Source: PLoS One. 2021 Oct 15;16(10):e0258656. doi: 10.1371/journal.pone.0258656 (PMC8519446; doi:10.1371/journal.pone.0258656)
Supplement: Attachment — Submitted filename: referee_report.pdf [file pone.0258656.s010.pdf]

<u>**Referee Report**</u>

Banned by the law, practiced by the society: The study of factors associated with dowry payments in Uttar Pradesh and Bihar, India

Summary
This paper looks at the correlates of the prevalence of dowry in UP and Bihar in India. It uses an adolescent survey conducted in these two states. Using a conditional correlation empirical setup, it documents important correlates such as education, wealth, and age at marriage, thereby aiming to contribute to the knowledge of dowry prevalence in India. There are some key areas in which this paper needs significant modifications. Following comments are aimed at highlighting those areas and providing suggestions wherever possible.

Comments:

1. While the goal of the paper is clearer, the contribution needs more work. Authors cite literature that has looked at the factors explaining cross-sectional differences in dowry practice and linking it to marriage market conditions and socio-economic factors. The variables authors look at are not too different from these factors and the contribution seems unclear. Authors need to clearly explain how their study is an addition/complementary to the existing work. In addition to that, the paper needs to document what gaps it is attempting to bridge in the literature. While doing that, a clear and coherent discussion of the existing literature is required. It is imperative to document the main findings of existing studies and any unexplored gaps. Finally, how this study aims to fill those gaps and what equips this study to look further than the existing literature. Also, how is the survey different from other larger-scale publicly available survey data such as IHDS and DHS? What is specific about this survey that allows for an exploration which would have not been possible otherwise (if so)?

2. This comment relates to the lack of mechanisms in this paper. The paper relies on existing literature and links their findings to the empirical analysis in this paper. However, this is not adequate in terms of exploring mechanisms. In absence of a non-causal setup, this becomes particularly difficult but no less important. Before linking the correlates to the theoretical work in the field (as cited by the authors), authors should try to explore the mechanisms of their findings using an empirical framework and the data used in this study.

    a) Regional or cluster level heterogeneities in explanatory variables can be utilized to explore the link between the prevalence of dowry and potential mechanisms (which can often be conflicting based on the findings of existing theoretical and empirical literature). These heterogeneities could be based on:
        I. Regional differences in education levels of marriageable girls and boys.
        II. Regional difference in age of marriage for both girls and boys.
        III. Regional differences in wealth.
        IV. Regional differences in caste mix. Homogeneity in caste mix may have different implications for dowry practice as compared to higher diversity in caste mix working through the channels of societal norms, culture, etc.

    b) Dowry prevalence is highly correlated with individual characteristics and regional or group characteristics. It can be thought of as a function of differences in the distribution of individual independent variables and the differences in groups and regions induced by

these independent variables. Authors should explore if they can decompose the observed effect into these categories using methods like Oaxaca-Blinder decomposition. This may go a long way in unmasking some important effects and put them in the context of existing literature.

c) After the empirical work exploring the channels that data allows for, existing literature on societal norms, culture, and prevalence of dowry can form a more organic link between the findings and possible explanations of the findings.

d) Authors can use other publicly available primary data such as DHS and IHDS to explore a much larger set of regional heterogeneities in relevant explanatory factors. This however is conditional on being able to link the regions (district etc.) in the survey to these other datasets.

3. Empirical work in this study needs improvement on multiple grounds. Some very important areas are below:

   a) The paper currently lacks the features of a representative conditional correlational analysis in the field. In the presence of a potential omitted variable bias and no direct measure in form of a causal framework to counter that, I would suggest the following:
      I. Testing the sensitivity of explanatory variables to addition and removal of other linked controls. All explanatory factors should be added sequentially and their sensitivity to the addition of further variables/controls should be documented before the direction and magnitude of their correlation to dowry prevalence can be discussed in results. For a few key variables such as household wealth, education, etc – their coefficient stability can be subjected to randomly dropping other regional and demographic controls.
      II. Explore the coefficient stability more formally using statistical exercises that provide bounds for the treatment effects, such as Oster (2016).

   b) Multiple correlates can be thought of as multiple hypotheses being tested. The coefficients should be subjected to tests for multiple hypotheses such as Benjamini-Hochberg correction.

   c) Discuss why a logistic distribution is more appropriate for this setup as compared to something like a linear probability model.

   d) Formally describe the model along with the variable construction. Are there any regional fixed effects? What is the level of clustering of standard errors?

   e) Results should be reported with the difference in coefficients and standard errors.

   f) Are there any sampling weights? They are more important in conditional correlations.
