# Supplementary material for: Banned by the law, practiced by the society: The study of factors associated with dowry payments among adolescent girls in Uttar Pradesh and Bihar, India
Source: PLoS One. 2021 Oct 15;16(10):e0258656. doi: 10.1371/journal.pone.0258656 (PMC8519446; doi:10.1371/journal.pone.0258656)
Supplement: Attachment — Submitted filename: Response to reviewers.docx [file pone.0258656.s008.docx]

**Reviewer Comments:**

**Overall comments**

1. This study identifies the factors associated with dowry among adolescent girls in UP and Bihar. The theoretical underpinnings of the study remain unclear. The Intro does not adequately present the rationale of conducting this study among adolescent in two Indian states. It does not explain the theories it alludes to and does not clarify the position of the authors. The Methods section does not elaborate on sample selection. The sub-section on statistical analysis leaves out many important details. For instance, it does not describe how wealth ranking index was constructed. Also, it does not provide any justification of pooing data from the two states and does not describe how pooling was done. The authors do not explain whether their model construction was guided by any theories. Selection of explanatory variables seemed ad hoc. No surprises await the reader in the Results section. The Discussion section seems disjointed and it does not flow logically.

**Response:** The introduction part has been modified as per given suggestions. Method section is also improved as per given suggestion.

**Detailed section by section comments**

2. The title of the study needs to mention adolescent girls.

**Response:** Thank you for the comment. Now adolescent girl is incorporated in the title.

3. The Abstract needs to be revisited after major revision of the manuscript has been made.

**Response:** Revised as per suggestion.

-------------------------------------------------------------------------------------------------------------

**Section:  Introduction:**

Page Number: 3

1. Comment: The Intro gives an impression that India represents a monolithic society, whereas it is widely known as not. The authors need to be conscious of and write about different customs not only by socioeconomic status, but also by caste, ethnicity and religion.

**Response:** The introduction section has been improved as suggested.

2 Text in the paper:     Historically, dowry served the fundamental purpose of inheritance for women as men were thought to inherit the family property in theIndian context [3]. Moreover, dowry has been seen as a way to compensate the groom’s family for the economic support they would provide to the new member of the family ….

Comment: The last two statements present two different concepts and cannot be presented as different aspects of the same phenomenon.

**Response:** The above-referenced sentence has now been followed by statement that better describe the meaning in text.

Text in the paper:     Furthermore, recent studies noted that the dowry system is prevalent across many cultures and is no longer is treated as a contribution towards a suitable beginning of the practical life of newly married couples [6].

Comment:  Please mention what the literature tells us about this transition. Also, be explicit about how dowry is treated now.

**Response:** The next paragraph after the sentence is describing the transition accordingly.

5. Text in the paper:     The concept of Sanskritization was proposed by eminent sociologist Srinivas in 1952, and many communities that never took dowry before started practicing dowry probably due to the phenomenon of Sanskritization [8].

Comment: Once you mention this please briefly describe what does Sanskritization mean and why would it lead to dowry practice.

**Response:** Authors feel that it is not important to describe ‘Sanskritization’ as this phenomenon is widely known and also not very much relevant to this manuscript.

6. Text in the paper:     Not only this, but studies have also noted that dowry payments have increased manifolds in India [10,11].

Comment:  Does this study represent all of India? If not, please put this finding in context.

**Response:** Yes, these studies present pan-India scenario. Therefore, there is no need to present further studies.

7.  Text in the paper:     A clear explanation for rising dowry payments is the marriage squeeze [12]. However, in his study, Anderson refuted the claims of any association between marriage squeeze and dowry payments [11]. Researchers unanimously agreed that the problem lies with gender inequality and female deprivation at every stage [7,13]

Comment: Please describe briefly what is marriage squeeze and why do you consider it a clear explanation for rising dowry. You seem to move from this position in the last sentence of the mentioned text. Please clarify your position explaining its justification.

**Response:** Marriage squeeze is tightness of marriage market. It can be Male as well as female marriage squeeze. A short description is provided in the text at relevant space.

8. Text in the paper:     Researchers unanimously agreed that the problem lies with gender inequality and female deprivation at every stage [7,13].

Comment:               At every stage of what?

**Response:** It means that every stage of life- from birth to childhood, and then further from childhood to adulthood and so on.

Page Number:          4

9. Text in the paper:     Despite acknowledging the problem of dowry widely, there is a paucity of empirical studies that systematically analyze the causes of dowry among adolescent girls in recent times.

Comment:  You are not investigating causes of dowry in this paper. So, this criticism is equally relevant for you as well. Suggest talking instead about correlates of dowry, which is your topic. You have not explained what justifies a focus on adolescent girls, whereas it is well known that dowry demand is usually lower for younger girls. Also, what justifies focusing on adolescent girls in an era when child marriage is reducing?

**Response:** Authors find this suggestion very relevant and accordingly changes have been made.

10. Text in the paper:     Given the growing concerns about the dowry's socioeconomic consequences, it is imperative to explore the dowry in India

Comment:   Please be more specific and say explicitly why you claim your study as important.

**Response:** The study is important because in India very limited data is available to conduct such type of study. Dowry related data is scarce in India and therefore this study becomes important.

11. Text in the paper:  While examining the causes of dowry among adolescent girls, this study contributes to the existing literature on marriage transactions in India.

Comment: You are not exploring the causes of dowry. Your method would not support you to draw any causal inference.

**Response:** Authors agreed with the reviewer and accordingly changes have been made.

**--------------------------------------------------------------------------------------------------------------**

**Section: Methods**

1. Please explain what drove selection of UP and Bihar as your studied sites. What are the characteristics of these two states that make them interesting for conducting such a study on dowry?

**Response:** comment incorporated. These two states have highest adolescent population in the country and age at marriage is also low; therefore, makes the study relevant in this regard.

2. I understand this is not the primary study. However, the distinction between the primary and secondary study has not been made clear in the ms.

**Response:** The study is secondar in nature.

**3.** Text in the paper:     The sample size for Uttar Pradesh and Bihar was 10,350 and 10,350 adolescents aged 10-19 years, respectively.

Comment: This is not an adequate description of sampling. Please elaborate on sample selection.

**Response:** comment incorporated.

**------------------------------------------------------------------------------------------------------------**

Section:                  Outcome variables

1. Text in the paper:     The question was framed as: “whether dowry paid at the time of marriage”? the response was coded as 0 means “no” and 1 means “yes.”

Comment: Are all dowry payments made at the time of the marriage? If not, you are risking miss- classifying the girls who paid a dowry after marriage as who had not paid dowry. If you don’t have the necessary data for addressing this issue please be more specific and say you will identify determinants of dowry payment at marriage rather than dowry per se.

**Response:** The question was framed as: “whether dowry paid at the time of marriage or later”? Therefore, captures the whole information.

----------------------------------------------------------------------------------------------------------------

Section: Explanatory variables

Page Number:          4

1. Comment: Please elaborate on how did you decide which variable to include as your explanatory variable?  Also, please mention how each of the variable was coded.

**Response:** comment incorporated.

Page Number:          5

2. Text in the paper:     Age at first cohabitation was recoded as never cohabited, less than legal age (<18 years), more than legal age (≤18 years).

Comment: The literature shows that age at marriage is associated with dowry. Your paper is about dowry paid at marriage. It is not clear what role age of cohabitation may have in this. Please present rationale for not including age at marriage, but including age at cohabitation as a explanatory variable.

**Response:** Age at marriage as a variable added.

3. Text in the paper:     Spousal education recoded both not educated, only husband educated, only wife educated, and both educated.

Comment: How relevant is no education category for the studied age category in India now? How many couples were in the category ‘both not educated’? Other studies showed an association between dowry and level of education. What makes you ignored that? I understand status inconsistency may be an important explanatory variable. But you could use a finer explanatory variable if you had not just a broad category ‘both educated’ category, but more categories within this broader category. My hunch is that there will be a lot of variation in couple education in this category. It won’t be wise to wipe out that variation.

**Response:** comment incorporated.

4. Text in the paper:     Working status was recoded as no and yes

Comment:               Please specify whose working status you are referring to. While education often discontinues with marriage and thus we can treat education as an attribute of the girl attained pre-marriage, we cannot have the same assumption for work or vocational training. Thus, without knowing exactly the temporal ordering it'd not be right to include these variables as explanatory variables in the model.

**Response:** comment incorporated.

5. Text in the paper:     Whether vocational training received or not was recoded not received and received

Comment:               Once again, whose vocational trainings are you referring to?

**Response:** comment incorporated.

6. Text in the paper:     Mother’s education was recoded as no education, 1-7, 8-9, and 10 and above years of education.

Comment:               Whose mother's education?

**Response:** comment incorporated.

7. Text in the paper:     Land ownership among in-laws was coded as no and yes.

Comment:               Who did you include among the in-laws? Does land ownership of the in-laws included in the study actually matters for decision-making about marital transaction? If you have data on amount of land owned it will be much better to introduce more categories of land ownership.

**Response:** comment incorporated. Amount of land data was not available in the study.

8. Text in the paper:     Caste was recoded as Scheduled Caste and Scheduled Tribe (SC/ST) and non-SC/ST

Comment:               Do inter-caste marriages take place? How did you deal with such marriages?

**Response:** Data on **inter**-caste marriages was not available.

9. Text in the paper:     Religion was recoded as Hindu and non-Hindu

Comment:               Do inter-religion marriages take place? Was there any in your sample? If so, how did you deal with such cases?

**Response:** Data on **inter**-religion marriages was not available.

10. Text in the paper:     Wealth index was recoded as poorest, poorer, middle, richer, and richest

Comment:               This description is inadequate. Please describe in detail how did you come up with wealth index.

**Response:** comment incorporated

----------------------------------------------------------------------------------------------------------

Section:                  Statistical analysis

1. Please explain why did you pool UP and Bihar data despite the fact that the sample was representative of each state. Are these two states so identical that the results would have been the same? How do you label the pooled data? How did you pool the data? Did you use any weights? Please add the details.

**Response:** comment incorporated.

2. Page Number:          5

Comment:               Didn't you conduct any descriptive analysis? Didn't you check any statistical difference? If you did, please mention them.

**Response: I**ncluded in table-2.

---------------------------------------------------------------------------------------------------------------

**Section:                     Results:**

1. Page Number:          5

Text in the paper:     Table 1.

Comment:               This Table confirms that you have actually conducted descriptive analysis.

**Response:** Thank you

2. Page Number:          6

Text in the paper:     About 65 percent of adolescent girls did not know their husbands before marriage, and interestingly, 63 percent of girls cohabited before the legal age

Comment:               The opposite has been shown in Table 1. By the way, what is so interesting about 65% of the girls not knowing their husbands and 63% cohabiting before the legal age (I guess) you mean marriage? Does India have a law regarding minimum age at cohabitation or is the law about minimum age at marriage? Please clarify this before making such comment. Please add age distribution of the girls and at marriage.

**Response:** comment incorporated.

3. Number:                 6

Comment:               It is unclear how wealth index was constructed. Distribution of the households by wealth index looks counter-intuitive. In LMICs it is usually expected that distribution of wealth will have a pyramid like structure with the poorest at the bottom and the richest at the top. As I understand, you must have pooled the two samples from UP and Bihar. Bihar is well-known as one of the poorest states in India. So, my hunch is, the distribution is likely to meet the usual expectation. However, this is not what is shown in Table 1. Please explain what is going on here.

**Response:** comment incorporated

4. Text in the paper:     Interestingly, paid dowry was significantly higher in marriages of girls not working (86%) or received vocational training (91%).

Comment:               Please clarify whether this working status and vocational training refers to pre-marital period or not. If not, you cannot conclude this.

**Response:** comment incorporated

5. Text in the paper:     Moreover, paid dowry was lower among the non-Hindu community (84.5%).

Comment:               This figure does not tell us that it is lower than among the Hindus. Please add the prevalence of dowry among the Hindus for making the difference clear to the readers.

**Response:** comment incorporated

6. Text in the paper:     Interestingly, the dowry was more prevalent in the richest households (88.7%).

Comment:               I have nothing against this finding. See the previous comment on wealth ranking. I am not convinced the index was correctly computed. I suggest you describe the analysis adequately, double check your analysis and then draw conclusions.

**Response:** comment incorporated

--------------------------------------------------------------------------------------------------------------

**Section:                     Discussion:**

1. Page Number:          10

Text in the paper:     Our study report that a girl knowingly her husband before marriage is less likely to pay dowry than those not known about the husband

Comment:               Please correct as "knowing".

**Response:** Thank you for the suggestion. Now we have replace by the word “knowing” in the manuscript.

2. Text in the paper:     This may be because the information about knowing each other is much better in a love match [20], and the practice of dowry is almost nonexistence in the case of love marriage [19,21].

Comment:               Please correct as "existent." Also, the sentence seems to suggest that knowing each other reduces dowry, but it is nt necessarily so.

**Response:** Thank you for the suggestion. Now we have replace by the word “existent”.

3. Text in the paper:     However, in an arranged marriage where information about each other is limited, the groom's quality is inferred through his education, associated with the dowry level [20].

Comment:               This is not what you found.

**Response:** I agree with the comment. This statement was cited from other study to support our finding.

4. Text in the paper:     The age of the bride is the main factor in marital negotiation, particularly in rural India.

Comment:               If you believe so, why did you not include age in your model?

**Response:** Thank you for the comment. In our study we have already include the age at marriage in the model.

5. Text in the paper:     The probable reason may be that a highly educated groom is more likely to marry late and more likely to marry a younger bride

Comment:               You do not need to speculate about this. You have the data to check this.

**Response:** Thank you for the comment. We now remove this sentence.

6. Text in the paper:     Specifically, a highly educated person is likely to have good qualities like commitment, sincerity, and loyalty.

Comment:  Who said so? Where is the evidence? If there is evidence please add citation.

**Response:** Thank you for the comment**.** We have reframed the statement for better understanding from the original source cited in the text.

7. Page Number:          11

Text in the paper:     It is generally believed that if women become more educated or employed, then dowry would be reduced or eliminated. However, our study reveals contrasting results as girl education does not impact reducing the practice of dowry. However, our study reveals contrasting results as girl education does not impact reducing the practice of dowry.

Comment:               Your point is about education and not about employment. So, don't bring up employment.

**Response:** Thank you for the comment. We now have removed the term employment.

**8.** Text in the paper:     Dalmia & Lawrence (2005) explained that the money transfer from brides and their families to grooms and their families does not decrease in the bride’s schooling [2].

Comment:               What does this mean?

**Response:** We have reframed this statement. Th actual meaning is that the amount of dowry or money paid by the bride’s family to the groom family does not reduce even though if bride is having a high level of education.

9. Page Number:          12

Text in the paper:     This regional difference in dowry payment could be explained by its different socio-cultural customs regarding dowry [2,10].

Comment:               Please don’t be vague. Spell out differences in which sociocultural customs explain regional differences in dowry.

**Response:** Thank you for the comment. We have elaborate more on this part to explain the socio-cultural practices which bring regional differences in dowry.

**10.** Text in the paper:     As India is a country with diverse social and cultural practices, dowry will vary with respect to their cultural norms.

Comment:               The section on limitations is too brief and leaves out many limitations that the study suffers from. This needs to be addressed. Some of these limitations may be gleaned from my comments.

**Response:** Thank you for the comment. We have include some more study limitations.

**--------------------------------------------------------------------------------------------------------------Section:                     Conclusion**

1. Page Number:          12

Text in the paper:     The study sought to explore the factors associated with the practice of dowry among adolescent girls in Bihar and Uttar Pradesh

Comment:               As these girls have little to do with dowry themselves, saying "practice of dowry among adolescent girls" does not sound right. Please rephrase.

**Response:** Thank you for the Comment. We now have rephrased the sentence.

**2.** Text in the paper:     It is evident from this study that the practice of dowry is still widespread, and the results show that increasing age, education, and household economic status of girls are associated with the likelihood of high dowry payment

Comment:               So, will marrying girls earlier, without educating them and living in poverty help reduce dowry?

**Response:** The point we want to address here is that if a girl is marrying at a later age, and even if she is well educated, still she have to pay more dowry. Specifically means that girl’s education does not influence much to reduce dowry payment. Also, it doesn’t mean that a girl from poor family need not pay dowry to the groom, what we mean is if a girl is belonging to a better economic household then the groom’s will more likely to asked for a higher dowry from the bride’s family.

3. Text in the paper:     However, significant causes are deeply rooted in social and cultural customs, which cannot be changed using laws.

Comment: What is your point about limitations here?

**Response:** The point here is that even though the Government of India has passed the Dowry Prohibition Act (1961) it is still not able to reduce or stop the practice of dowry. This is because dowry is deeply rooted within the Indian culture, and its practice is still prevalent in the Indian society especially in the rural areas where people strictly fallows the culture.

4. Text in the paper:     Our study suggests that only the socio-economic development of women will not protect her from the dowry system

Comment:               Please indicate clearly which finding/s from this paper suggest/s this.

**Response:** Women still have to pay dowry even though she belong to the better economic status (Wealth Index variable), and even she is educated but not her husband (Spousal education variable)

5. Text in the paper:     A community-level approach is necessary to develop their level of understanding and awareness to understand the negative impact of such an evil custom.

Comment:               Which of your result/s suggest/s this? Who are you referring to here? What makes you assume that "they" lack awareness and understating of the negative impact of dowry?

**Response:** Thank you for the comment. Like we have mentioned before that in India dowry practice is attached to the cultural believe. So to make them understand about the negative impact of dowry, awareness programme at the community level is important to change the community attitude and make them rethink about the dowry system.

6. Text in the paper:     There are some unique and exceptional causes regarding dowry that need to be considered during policy development.

Comment:  Please be specific and tell the reader what exactly are you proposing.   Here we are switching again to cause of dowry.

**Response:** Even thought the Indian Dowry Protection Act prohibits the practice of dowry, but dowry practice is still continuing in India. So while framing anti dowry policy or law it is important to consider the unique causes of dowry such like culture or religious believe.

**REVIEWER 2**

The authors have attempted to study the risk factors for Dowry demand based on the survey that was done UP and Bihar. The authors have attempted to provide good information about the risk factors. Though this is a good attempt the following comments would be useful to improve the presentation of the manuscript.

1. First they need to provide the overall prevalence of Dowry with 95% CI and separately for UP and Bihar, plus Rural and Urban areas.

**Response:** comment incorporated. Added in table-2.

2. What the sampling process involved. There is no reference available to read about the sampling methods available. Do we have any report about this survey?

**Response:** comment incorporated.

3. Whom did you interview? Is this the girl ? or parents of the girl?

**Response:** Girls**.**

4. The Logistics regression is a well known method and very widely used and therefore, they do not have to provide the model in the method section.

**Response:** Adjusted model was in table-3.

5. The authors are ignorant about presenting the Risk Factors data in table. A simple table of data was stretched into 3 tables. They need to review some papers in the literature and follow the style. They need to review some risk factors papers and follow the style. For example,
REF. Journal of Biosocial Science Volume 47 Issue 6 2015, DOWRY DEMAND AND HARASSMENT: PREVALENCE AND RISK FACTORS...

**Response:** comment incorporated.

6. The conceptual frame work is missing.

**Response:** Added**.**

----------------------------------------------------------------------------------------------------------------
