# Supplementary material for: Banned by the law, practiced by the society: The study of factors associated with dowry payments among adolescent girls in Uttar Pradesh and Bihar, India
Source: PLoS One. 2021 Oct 15;16(10):e0258656. doi: 10.1371/journal.pone.0258656 (PMC8519446; doi:10.1371/journal.pone.0258656)
Supplement: Attachment — Submitted filename: Response to editor.docx [file pone.0258656.s012.docx]

1. Elaborate on the sample selection procedure – what steps were followed.

**Response**: Comment incorporated (Figure-1).

2. Robustness on LPM must be added as a table.

**Response**: Comment incorporated.

3. Please use Oster (2016) when you do the LPM (linear probability model).

**Response**: Comment incorporated.

4. I will like to see a table comparing the basic descriptive statistics with IHDS or REDS (I will prefer REDS over IHDS).

**Response**: The authors cannot use IHDS or REDS data for the following issues.

(a): IHDS data gives estimates at national level and therefore, state-level analysis cannot be performed. It is not a good idea to perform state-level analysis as the data is not state-representative. In this study, we have used UDAYA data specifically designed for Uttar Pradesh and Bihar. Conceptually, it is not feasible to compare the two datasets.

(b): The UDAYA data we used is from 2016. However, the latest REDS data is from 2006. Therefore, the descriptive analysis may not be that useful. So, authors feel that it won’t be a good idea to generate a table (using IHDS/REDS data) for bivariate association.
