# Supplementary material for: Banned by the law, practiced by the society: The study of factors associated with dowry payments among adolescent girls in Uttar Pradesh and Bihar, India
Source: PLoS One. 2021 Oct 15;16(10):e0258656. doi: 10.1371/journal.pone.0258656 (PMC8519446; doi:10.1371/journal.pone.0258656)
Supplement: Attachment — Submitted filename: Response to editor.docx [file pone.0258656.s013.docx]

**Editor’s comments:**

1. I cannot find the Oster (2017) table.

**Response:** Dear Editor, We are really sorry as we are unable to understand the Oster (2017) table. We tried our best but could not prepare the required table. We also took help from some of the individuals/researchers, however, none of them could come out to our rescue as everyone failed to understand the concept. We are really sorry on our part as we were unable to understand the relevant methodology and therefore, we are not including Oster (2017) table. If the table is absolute necessity, we would seek the editor’s guidance in this regard.

2. Please have detailed notes in all tables -- some have no notes.

**Response:** All the tables have been updated as suggested.

3. Have proper labels for all variables used in the tables.

**Response:** Labels have been formatted as per the suggestion.

4. IHDS (2005 and 2011) can be used for state level analysis as its representative (so is REDS). Although its an older data, it important to show the table.

**Response:** Authors have used IHDS data to show the relevant information. Please find the table attached to this response.

| Table : Socio-economic characteristics of married girls 15-19, 2004-05 and 2011-12 | | | | |
| --- | --- | --- | --- | --- |
| **Background characteristics** | **IHDS 2004-05** | | **IHDS II 2011-12** | |
|  | **Sample** | **Percentage** | **Sample** | **Percentage** |
| **Gift paid at the time of marriage** |  |  |  |  |
| No | 0 | 0 | N/A | N/A |
| Yes | 347 | 100 | N/A | N/A |
| **Age at marriage (in years)** |  |  |  |  |
| Less than 15 | 124 | 35.7 | 70 | 30.0 |
| 15 and more | 223 | 64.3 | 163 | 70.0 |
| **Literate** |  |  |  |  |
| No | 196 | 57.0 | 79 | 33.9 |
| Yes | 148 | 43.0 | 154 | 66.1 |
| **Working status** |  |  |  |  |
| No | 256 | 73.78 | 184 | 79.0 |
| Yes | 91 | 26.22 | 49 | 21.0 |
| **Caste** |  |  |  |  |
| SC/ST | 95 | 27.4 | 67 | 28.8 |
| Non-SC/ST | 252 | 72.6 | 166 | 71.2 |
| **Religion** |  |  |  |  |
| Hindu | 293 | 84.4 | 184 | 79.0 |
| Non-Hindu | 54 | 15.6 | 49 | 21.0 |
| **Wealth index** |  |  |  |  |
| Poorest | 97 | 28.0 | 47 | 20.2 |
| Poorer | 61 | 17.6 | 47 | 20.2 |
| Middle | 77 | 22.2 | 46 | 19.7 |
| Richer | 59 | 17.0 | 47 | 20.2 |
| Richest | 53 | 15.3 | 46 | 19.7 |
| **Residence** |  |  |  |  |
| Urban | 66 | 19.0 | 37 | 15.9 |
| Rural | 281 | 81.0 | 196 | 84.1 |
| **State** |  |  |  |  |
| Uttar Pradesh | 245 | 70.6 | 155 | 66.5 |
| Bihar | 102 | 29.4 | 78 | 33.5 |
| **Total** | 347 | 100 | 233 | 100 |
| N/A: Data Not available;  Dowry is assessed from the question "Generally in your community for a family like yours, what are the kind of things that are given as gifts at the time of the daughter's marriage?" Gold, Silver, Land, Car, Scooter/motorcycle, TV, Fridge, Furniture, Pressure Cooker, Utensils, Mixer grinder, Bedding/mattress, Watch, Bicycle, Sewing machine, Livestock, Tractor and Cash. | | | | |

* Please note that the dowry prevalence is 100 percent for the year 2004-05. The question related to dowry asked in IHDS was different than what was asked in UDAYA data.

Furthermore, authors could not utilize REDS data as the relevant data were not provided to the authors. We requested to the concerned authority for data access, however, we were told that they only grant access in certain conditions that are needed to be fulfilled by the institution. They only grant access to the institution and from institution only we can borrow data after receiving proper IRB approval which was not feasible in this case.

This is what they said:

To obtain cross-walk to village identifiers and/or previous survey rounds or any of the 2006 data, the investigator must obtain IRB approval from their respective university or institution. The IRB-approved proposal should indicate the following:

a) that statistical identification of households or villages is possible with the secure data

b) that linked data and cross-walks will be kept in a secure environment

c) names of all investigators and research assistants with access to the linked data

d) that linked data will only be used on projects with IRB approval

e) that the linked data or the cross-walk cannot be provided to investigators or researchers

not specifically identified in (c)

f) that publications and presentations must not reveal village or individual level identifiers.

I cannot proceed unless you address these 4 points. Also, I found the tables quite sloppy.

**Response:** We have edited the tables as per given suggestions. Furthermore, we tried to incorporate all the comments suggested by the editor except that Oster (2017) related comment. We seek advice from editor on this issue.
