# Supplementary material for: Banned by the law, practiced by the society: The study of factors associated with dowry payments among adolescent girls in Uttar Pradesh and Bihar, India
Source: PLoS One. 2021 Oct 15;16(10):e0258656. doi: 10.1371/journal.pone.0258656 (PMC8519446; doi:10.1371/journal.pone.0258656)
Supplement: Attachment — Submitted filename: Response Letter.docx [file pone.0258656.s014.docx]

**MANUSCRIPT TITLE:** Banned by the law, practiced by the society: The study of factors associated with dowry payments among adolescent girls in Uttar Pradesh and Bihar, India

**MANUSCRIPT ID:** PONE-D-21-04546R3

Respected editor,

Thank you for giving us the opportunity of submitting an improved version of our manuscript for publication in the PLOS One. We are highly grateful to receive your insightful comments and suggestions. We appreciate the time and effort that you have put forward to provide valuable feedback that has significantly improved our paper. Kindly note that we have incorporated the changes that were suggested. The modifications have been shown using track changes within the revised manuscript. Please see below for a point-by-point response to each of the comments and suggestions.

Hoping that you and your family members are safe and sound in these challenging times,

Yours Sincerely,

Authors

**EDITOR COMMENTS:**

1. Journal Requirements: Please review your reference list to ensure that it is complete and correct. If you have cited papers that have been retracted, please include the rationale for doing so in the manuscript text, or remove these references and replace them with relevant current references. Any changes to the reference list should be mentioned in the rebuttal letter that accompanies your revised manuscript. If you need to cite a retracted article, indicate the article’s retracted status in the References list and also include a citation and full reference for the retraction notice.

Response: Thank you for pointing this out. Comment has been incorporated.

2. Additional Editor Comments (if provided): Please see: https://emilyoster.net/research/. On this page look for JOURNAL OF BUSINESS ECONOMICS AND STATISTICS, JUNE, 2019, Unobservable Selection and Coefficient Stability: Theory and Validation. They provide the do file to undertake the exercise. W/o this paper I cannot proceed.

Response: Dear Editor thank you very much for the suggestion. Based on your comment, we proceeded for stability check of the study coefficients after reading Emily Oster’s research article. The results for the same has been shown in Table 4 of the manuscript. The methodology has been discussed in the “statistical methods” section as shown below:

*Next, we check the stability of the regression coefficients and their sensitivity to selection bias using standard methods. We obtain bias-adjusted coefficients and calculate the absolute deviation from the non-bias-adjusted regression estimates to understand the extent of bias. Further, we calculate Oster's δ, whose value higher than one would indicate that the regression coefficients are insensitive to omitted variable bias and variable selection bias. All estimates were obtained with the assumption that the bias-adjusted model would explain 1.3 times variation in dowry payment status compared to the non-bias-adjusted model. The statistical analyses for coefficient stability check were performed using the psacalc command by estimating linear probability models in STATA.*

The results for the stability check are discussed in the “Results” section (Page 13):

*Table 4 gives the results of the coefficient stability check of the explanatory variables of dowry payment among female adolescents. From the bias-adjusted estimates (see column 8), we observe that the multivariable association between husband familiar before marriage, age at marriage, spousal education, wealth index, residence and state with dowry payment is statically significant (at 5% level) and lies in the same direction as the uncontrolled estimates. Moreover, from the difference shown in column 10, we can say that the bias-adjusted and non-bias-adjusted regression coefficients are similar. However, Oster's delta revealed that the statistically significant multivariable association of age at marriage and state with dowry payment suffers from omitted-variable and selection bias.*

Further, we have added the following line as study limitation in the discussion section (Page 18):

*Although the coefficient stability check revealed that the majority of the explanatory characteristics are insensitive to omitted-variable and selection bias, the results for age at marriage and state need to be interpreted with caution.*
