# Supplementary material for: Banned by the law, practiced by the society: The study of factors associated with dowry payments among adolescent girls in Uttar Pradesh and Bihar, India
Source: PLoS One. 2021 Oct 15;16(10):e0258656. doi: 10.1371/journal.pone.0258656 (PMC8519446; doi:10.1371/journal.pone.0258656)
Supplement: Attachment — Submitted filename: Referee Report-Dowry.docx [file pone.0258656.s009.docx]

**Referee Report on "Banned by the law, practiced by the society: The study of factors associated with dowry payments in Uttar Pradesh and Bihar, India"**

*Summary & General Evaluation*

Dowries are wealth transfers from the bride’s family to the groom or groom’s family paid at the time of the wedding. In India, the practice of dowry payment is highly prevalent and typically several times the yearly household income, despite being illegal since 1961. This paper studies the factors associated with a dowry payment using data from the Understanding the Lives of Adolescents and Young Adults (UDAYA) project, consisting of 5206 married adolescent girls from two states in India, Utter Pradesh, and Bihar.

Using logistic regression analysis, the authors document associations between the practice of dowry payment and various socio-economic factors such as the age of the girl, education of the girl, household socioeconomic status, spousal education and, spousal age gap, to name a few.

Let me start by saying that I find the exercise of establishing correlations between dowry payments and important socio-economic variables worthwhile, and the paper definitely furthers our understanding of the possible determinants of the decision to pay a dowry along with confirming some of the previous findings in the literature. However, in its current version, the paper could be significantly improved in terms analysis and writing. Below I summarize my main comments.

*Main Comments*

1. Data and Regression Analysis.  The following are my specific concerns with the data and analysis in the paper (The main text from the article is in blue with quotations):
a) "The sample size for this study was 5,206 adolescent girls who were married at the time of the survey" – The original sample is 10,350 adolescents from Utter Pradesh and 10,350 adolescents from Bihar; from this, you select 5206 adolescents. Please explain the sample selection procedure.
b) "Wealth index was recoded as poorest, poorer, middle, richer, and richest. The survey measured household economic status, using a wealth index composed of household asset data on ownership of selected durable goods, including means of transportation, as well as data on access to a number of amenities. The wealth index was constructed by allocating" - Doing a Principal Component Analysis on the variables and, creating a wealth component would be preferred.
c) "This is consistent with our study's findings, where the girls from the wealthier family were more likely to pay the dowry at marriage." – I am not sure what variable are you using for natal family wealth?
d) Since your sample by deﬁnition only includes girls aged 15-19. The fraction of girls who are legally going to marry above the legal age of 18 is by construction going to be small. The authors should mention this point in the paper.
e) $\pi$ needs to defined precisely in the specified logistic regression.
f) As a robustness check, the authors could see if these correlations hold true using a linear probability model instead of logistic regression.
2. Relation to existing works. The authors could have been more thorough in writing this paper, especially in citing relevant literature while explaining the main ﬁndings. There is extensive theoretical literature in economics on the emergence and the existence of dowries. In the introduction, while motivating the presence of dowries in societies like India, the authors cite some of the papers such as Anderson (2003) but miss out on some critical articles like Anderson and Bidner (2015); Botticini and Siow (2003).

Similarly, there is a growing empirical literature studying the determinants of dowry payments that have carefully analyzed questions related to this paper’s main ﬁndings. I recommend the authors carefully review the following articles:

a) Chiplunkar and Weaver (2019) carefully document the transition of dowry payments in India using the 1999 wave of the ARIS-REDS data and test which theories about dowry inﬂation are consistent with the data which are not. I highly recommend the authors to read this paper thoroughly. For example, the authors in the introduction talk about the Sanskritization theory, Chiplunkar and Weaver (2019) show that this theory cannot explain dowry inﬂation. Similarly, they also ﬁnd that the REDS data offers limited support to the marriage squeeze hypothesis.
b) Edlund (1999): The author also studies the hedonic regressions of dowry on bridal traits. However, she looks at actual magnitude dowry payments, different from this paper that looks at dowry payments on the extensive margin. A couple of sentences comparing results in this paper to yours will be beneﬁcial.
c) Arunachalam and Logan (2016) is also a related paper.
3. Exposition and takeaways. The discussion and the conclusion section need to significantly re-written for clarity. The authors make a series of claims in the discussion section that require a relevant citation. Similarly, the conclusion section can also be reworded in line with the main contribution of the paper. I list some of the specific instances below (The main text from the article is in blue with quotations):
a) "Researchers unanimously agreed that the problem lies with gender inequality and female deprivation at every stage" - This sentence needs to be reworded for clarity, and relevant literature that has documented the relationship between dowry payment and gender inequality at different stages of a woman’s life needs to be cited (Alfano, 2017; Bhalotra et al., 2020; Bloch and Rao, 2002; Zhang and Chan, 1999). Further, the claim that dowries are associated with female deprivation at every stage is not supported by the existing literature (Zhang and Chan, 1999).
b) "Despite acknowledging the problem of dowry widely, there is a paucity of empirical studies that systematically analyze the correlates of dowry among adolescent girls in recent times." - The authors need to cite relevant papers here.
c) "However, with the transition of time, the practice of dowry is becoming mandatory, and the demand for a higher dowry becomes a burden to the bride’s family." - There is a shift in dowries from a stridhan to a groom-price (Srinivas, 1984), but what is the evidence that the practice is becoming more mandatory?
d) "Finally, national representative data will be helpful for further study to understand the scenario of dowry practice in India, as because India is a country with diverse social and cultural practices, dowry will vary with respect to their cultural norms." - Rural Economic and Demographic Survey (REDS) of India is a nationally representative survey that contains dowry information.
e) "Instead, a massive social reform and action are urgently required to stop the practice and change their attitude about the system. A community-level approach is necessary to develop their level of understanding and awareness to understand the negative impact of such an evil custom. Simultaneously, it is necessary to restructure the existing dowry prohibition law to make it more effective. There are some unique and exceptional causes regarding dowry that need to be considered during policy development." - This goes well beyond the scope of the paper. The paper is documenting interesting correlations in a unique dataset between dowry payment and socio-economic characteristics. The conclusion should be about these associations.

*Other comments*

a) "However, in his study, Anderson refuted the claims of any association between marriage squeeze and dowry payments [11]." - This sentence has a notable typo; it should be in her study.
b) The paper in its current version has several typos and grammatical errors. The references section needs to be edited, too; please follow one reference style consistently throughout the paper.
